# A patienthood that transcends the patient: An analysis of patient research partners’ narratives of involvement in a Canadian arthritis patient advisory board

**DOI:** 10.1177/13558196231197288

**Published:** 2023-08-26

**Authors:** Graham G Macdonald, Jenny Leese, Alison M Hoens, Sheila Kerr, Wendy Lum, Lianne Gulka, Laura Nimmon, Linda C Li

**Affiliations:** 1 Graduate Programs in Rehabilitation Sciences, 8166 University of British Columbia, Vancouver, British Columbia, Canada; 2 School of Epidemiology and Public Health, 6363 University of Ottawa, Ottawa, Ontario, Canada; 3 Department of Physical Therapy, 8166 University of British Columbia, Vancouver, British Columbia, Canada; 4 Arthritis Patient Advisory Board, 258853 Arthritis Research Canada, Vancouver, British Columbia, Canada; 5 Department of Occupational Science and Occupational Therapy, 8166 University of British Columbia, Vancouver, British Columbia, Canada

**Keywords:** Patient engagement in research, patient journey, civic patienthood

## Abstract

**Objectives:**

Incorporating the perspectives of patients and public into the conduct of research has the potential to make scientific research more democratic. This paper explores how being a patient partner on an arthritis patient advisory board shapes the patienthood of a person living with arthritis.

**Methods:**

An analysis was undertaken of the narratives of 22 patient research partners interviewed about their experiences on the Arthritis Patient Advisory Board (APAB), based in Vancouver, Canada.

**Results:**

Participants’ motivations to become involved in APAB stemmed largely from their desire to change their relationship with their condition. APAB was a living collective project in which participants invested their hope, both for their own lives as patients and for others with the disease.

**Conclusions:**

Our findings highlight how the journeys of patient partners connect and integrate seemingly disparate conceptions of what it means to be a patient. One’s experience as a clinical ‘patient’ transforms into the broader notion of civic patienthood.

## Introduction

The involvement of the public and patients with chronic diseases as partners in health research has evolved over the last decade, with increasing emphasis on addressing the power structures inherent in scientific knowledge production, dissemination and implementation.^1^ Arthritis research is a field that spans a broad array of rheumatic disease groups, and has witnessed particularly dynamic patient involvement over the past two decades. The field has pioneered methods of involvement that are implemented and adapted as templates in other fields.^2^ In addition to aims like improving the quality of research and its relevance to patients, the process of incorporating the perspectives of patients and public into the conduct of research has the potential to make scientific research more democratic. The democratization of scientific research implies a redistribution of power to the voices and perspectives of the public, which may previously have been marginalized by various systems and institutions.^3,4^ Patient partners have been at the forefront of this process of democratization,^5,6^ and their participation indicates a field with complex power relations.^7–12^ Some have commented how it is difficult to conceptualize patient voice or perspective in a way that can authentically represent the complexity it contains.^13–15^

Many have noted that the construction of ‘patient voice’ or perspective is a normative project that is political in the sense that it seeks to change power dynamics.^13,16–19^ It has been pointed out that *any* ‘patient perspective’ requires a ‘patient experience’ as its basis.^19^ We borrow the concept of *patienthood* from Landzelius^20^ to refer to the experiences of being and processes of becoming a patient. Patienthood provides us a way to think of ‘the patient’ in a way that is in tune with the lived-in reality of people who have experienced being a patient, rather than as a status conferred upon an individual by an institution. Further, it allows us to talk about the evolutions, transformations, and differences inherent to that experience while maintaining a logical connection between them.

As such, this paper aims to explore how being a patient partner on a patient advisory board shapes the patienthood of a person living with illness – that is, how it affects their relationship to their health and illness.^21,22^ We examine what we have labeled ‘horizons’ of patient partners’ narratives of their involvement in research, and of their patienthood in general. Drawn from the philosophy of Hans-Georg Gadamer, ‘horizon’ is another way to conceptualize how the limits or boundaries of an understanding can change, fuse and grow.^23^ Put simply, one’s horizon is the range one perceives or understands.^24^ We also consider ‘horizon’ to imply not just an intellectual understanding but also an ethical understanding of one’s responsibility and power in a given situation. For example, a personal or individualistic horizon would imply that the power and responsibility to deal with one’s situation lies within oneself. Other horizons may integrate social, cultural, and political factors into an assessment of the power and responsibility in a given situation and conceive of the role of the individual as part of a collective. One’s horizon provides a framework for problem-solving, but also the ethical context in which that problem-solving takes place.

Our paper seeks to elaborate the complexity of patient experiences in research based on the perspectives of patient partners on a research advisory board, the Arthritis Patient Advisory Board (APAB), based in Vancouver, Canada. The APAB is a group of volunteers with at least one form of arthritis, who collaborate with scientists at a research center, Arthritis Research Canada, and serve as a bridge in disseminating research to the public. Many of these volunteers have also been involved in research as participants, though this is not required of them to join the APAB. As research partners, APAB members participate in identifying research topics, preparing grant applications, shaping the research design, recruiting participants, interpreting findings, co-authoring scientific papers, writing lay summaries, and/or attending or co-presenting at conferences. Furthermore, they mentor other patients, as well as researchers, trainees and research staff, in patient engagement in research.

At the time of the data collection, the APAB consisted of 21 members and 12 emeritus members (28 women, five men). The majority of APAB members reside in [region 1] (in urban and rural areas), one member is based in [region 2] and five live in [region 3]. Most APAB members had been involved in at least three research projects led by Arthritis Research Canada scientists, while long-term members had been involved in up to 20 projects.

## Methods

### Study design

This paper derives from a secondary analysis of data from a previous study ([redacted]). The following section includes the description of the sampling and interview methods of the previous study that are relevant and apply to this paper. The dataset used by both studies is the same. This dataset consisted of 22 semi-structured interviews with patient research partners at the APAB. The interviews focus broadly on their experiences as patient partners, revealing patient partners’ general experiences of involvement in the APAB.

The research team in both the original study and this paper consisted of four researchers and four patient partners from APAB.

### Participants

Eligible participants were past or present members of APAB. From August to November 2015, they were invited to participate in an interview via an email and word of mouth. Interested individuals contacted the lead research trainees (either by email or phone), who provided details about the study and screened for eligibility. Informed consent was obtained from all participants.

### Interviews and dataset

The previous study from which this secondary analysis derived consisted of semi-structured interviews. These were conducted with a convenience sample of past and present APAB members with varying degrees of experience in engaging in research. In 2015–2016, 22 participants were recruited, 21 (95%) were female, ages ranged from 26 to 68 years, and time spent as a patient research partner ranged from 1 month to 10 years. Thirteen (59%) had inflammatory arthritis, five (23%) had osteoarthritis, and four (18%) had both.

The interview guide was devised in partnership with APAB patient partners. ([redacted]) Interviews were conducted primarily by one of the authors ([redacted]) with help from a research assistant. These were done at a time and place convenient for the participant (e.g. [research center], the participant’s home or a café), or over the phone. The interview guide (see S1 in the online supplement) was organized into three separate but overlapping sections: (1) experiences/benefits/downsides of being a patient engaging in research; (2) interactions with researchers; and (3) perspectives on APAB’s development. Open-ended questions were asked by interviewers, and probes and prompts used for elaboration. In order to support participants generating their own thoughts on what was important based on their experience, specific feedback on published findings in the field of patient engagement in research was not sought. The interviews (lasting approximately between 30 and 90 min) were audiotaped to ensure accuracy.

Participants were known to each other, so care was taken to de-identify transcripts in advance of inviting participants to review and modify the transcript of their interview, and provide permission for their transcript to be used for analysis. Participants were also known to the research team that carried out the data collection. The team included patient partners from the organization under study, who were also interview participants in this study (co-authors WL, SK, and AH were all interviewed for this study). The patient partner co-authors were not involved in conducting the interviews of their fellow APAB members. As such, participants were only ever referred to pseudonymously, all pseudonyms were changed at least once.

A memorandum of understanding was developed to represent an agreement between APAB patient partners and researchers at Arthritis Research Canada to collectively own all data (subject to a participant’s approval), results and research outputs generated by the project. Patient and academic partners corresponded frequently by email throughout each phase of the research cycle (preparatory, execution and translation), and held bi-annual progress meetings (attended in-person or remotely).

### Data analysis

Academic personnel on the research team changed somewhat for this secondary analysis, but all four patient partners from the primary study agreed to join the research team for the secondary study. The core methodological difference between this analysis and the previous study was that instead of treating the data as an aggregate of participants’ experience, attention was given to the individual ‘narrative’ within each participant’s interview. The following addresses only the analytic approach applied to this paper and not the previous study.

An iterative approach using constant comparative methods was used to analyze the data. Our methodology draws on constructivist Grounded Theory,^25^ as well as narrative analysis.^26^ No preselected codes were identified prior to data analysis. The first round of coding focused on the individual ‘narratives’ of each of the 22 participants. By narratives, we mean to say we were not only interested in the content of what they were saying about patient engagement in research, but also the form. Attention to this form of ‘personal narrative’ gave us context against which we could draw significance from *why* and *how* things were said, rather than just the general content. Interesting quotes, anecdotes, and experiences were not simply picked from a wider pool to prove thematic points – they were first analyzed in the context of how and why the participant brought it up. The second round of coding consisted of a pattern analysis where the ‘individual narratives’ were compared and contrasted with each other. In a third stage of analysis, we considered what sort of relationships to illness were being described in participants’ narratives. In this latter stage, our analysis followed how those relationships to illness evolved, transformed, and generated ethical questions.

Coding was carried out predominantly via paper-based methods, and NVivo 10 was used for storage and management. Peer checking and member checking enhanced the rigor of our analysis. We consulted with study patient partners on the findings of this analysis to see if the analysis resonated with their experience. The process took place between 2017 and 2022, including a formal research meeting in which patient partners reviewed study findings, as well as reviews of multiple drafts of the manuscript. Feedback from the patient partners during meetings was recorded in meeting notes. Both written and verbal feedback from the patient partners were integrated into the analysis of the findings. Extensive analytic notes and memos were taken, and drafts of previous analyses were preserved to track the development of thinking on the study data.

S2 in the online supplement gives a timeline diagram. This provides a bird’s eye view of five iterations of this project, showing for each iteration: (1) the general thrust of the work at the time; (2) themes of memos and feedback; and (3) the resolution or product resulting from reflection on the feedback and memos.

## Results

Our analysis revealed the evolution of participants’ horizons in their ‘patient journey’ – that is, a person’s experience related to being a patient or living with an illness. We focus on three aspects of this journey: (1) Motivations and purposes, which addresses why participants got involved in research as patient partners and what it meant to them; (2) challenges and resolutions, which concerns how participants encountered and dealt with difficulties in their journeys as patient partners; and (3) reflection and re-orientation, which deals with how participants’ ‘patienthood’ comes to mean something different in the context of being a research partner.

### Motivation and purpose

For our participants, a common motivation to join APAB was the desire to learn about arthritis and how to manage it. This was often framed as a matter of taking control of the illness. Deka explained that being part of the APAB helped her deal with the ‘loss of control of your body and your life’ engendered by chronic illness. Heather explained the stark reality of having arthritis:

You have a chronic and crippling disease … it’s sort of the guardian at the gate. It shapes and interferes with and constrains your relationships with the world.

Becoming well-versed in the language and concepts of their disease was one way to deal with this guardian, and it often led participants toward research and researchers. As Jan explained:

It is important for me to ask the right questions in order to get the right answer, so I prefer to ask the questions myself to the researcher. It is also very important for me to get the firsthand information from the researchers themselves…I could bring back this information to my doctor with confidence.

Many participants framed their search for answers as being driven by a desire to help others in a similar situation and, in doing so, help themselves. As Jessica explained:

There’s an element of altruism that you … want to help others because you know what the struggle feels like for yourself, and there’s an inherent sense of value that your contributions may make the path a little easier for other people that are similarly having a challenging journey. So, it makes you feel like there’s some more purpose in what you’re experiencing.

Similarly, Jan said:

I’m learning from the other members some ideas that I could use to help myself with my disease. I’m happy to contribute and be part of the solution that could help others who are suffering with the disease. It’s a very fulfilling feeling.

And Madeline said:

The fact that I will give of my time, my energy, my thinking, my problem-solving … [to] benefit somebody, even if it’s 10 years from now. That to me is worth it.

Many who became involved in research did so because health care was not oriented to their needs as people with arthritis, and they wished to do something about that. Heather said she joined the APAB:

To articulate what we needed, since no one had ever asked us … People don’t ask people with arthritis, ‘What do you need?’, they *tell* you what you need.

Likewise, Chloe described some assumptions made in research:

Some of the conclusions I think are a bit unfair towards the patients. They say that this is, as a lifestyle, is too difficult, but they’re writing off the possibility that we are hugely invested in this. This is our life and, yes, it may be very difficult, but I would like the choice … And I know there’s not as much funding to go towards studies which don’t include a prescription medication.

Many participants became aware of other health care issues that also needed to be addressed. As Hannah put it:

I’ve gotten involved in a lot of different things, through wanting things to be different for patients in the health care system*.*

### Challenges and resolutions

Participants detailed their struggles with various aspects of becoming involved in research. Many spoke of a steep learning curve and self-doubt about their ability to make a valuable contribution. Laura described the common experience of being overwhelmed by information and trying to keep up in her first few APAB meetings:

I didn’t get myself in projects yet because I was still learning. I didn’t really say much the first little while … but it wasn’t that I wasn’t thinking, I was constantly thinking and trying to absorb.

Gaining a sense of familiarity or mastery over the subject matter was not enough for most participants to silence the self-doubt that came with being a patient research partner. Hannah described feeling like a ‘fraud’ because:

Most people who do research have their Master’s or their PhD or they’re working on Post-Doctoral projects. And I still am sitting here and I have no badge … I have nothing except my life experience that qualifies me to do research.

Victoria expressed a similar sense of self-doubt but offered a clear resolution to that. She said those around her made her feel like her life experience as a person with arthritis allowed her to make a valuable contribution:

I remember … looking around this table and saying, ‘Am I the only one without a degree and who am I?’ But by the same token, [I was] already feeling like I had contributed, so I felt like I was a member. I felt very included.

Sensei connected this feeling of self-doubt, and difficulty of processing an overwhelming amount of information, to a sense of vulnerability that can be felt by newcomers:

Nobody likes to be in a vulnerable position of not knowing what they’re doing ... Some people have a great deal of difficulty, they may want to [ask questions] but they have a hard time.

Some participants detailed difficulties in becoming more open and speaking freely about their illness, as well as confronting it. Sarah felt very uncomfortable with sharing about her illness, or even thinking too deeply about it, saying: ‘I’ve never talked about my disease, never’ and ‘my way of dealing with arthritis has been avoidance’. But she described her involvement on the APAB as a process of ‘overcoming’ such issues and helped her ‘come out of her shell’.

Heather had less trouble making her arthritis a matter of public concern, but through the APAB learned that many people with arthritis are reluctant to publicly reveal that they have the disease:

A lot of people don’t want to be identified as having arthritis because they’re afraid it will impinge their social life, their work, their career opportunities.

Participants emphasized the importance of group solidarity and mentorship in nurturing them and creating an environment and culture where patient partners could find confidence and overcome their self-doubt. As Victoria recounted:

Just knowing that you have a group behind you, knowing that if you’ve had a bad day and you’re not sure if you’re going to be able to sit through a whole meeting - you’re going to have to stand - that nobody’s going to look at you like you’re interrupting a meeting. Knowing that somebody’s going to email you and say, ‘Are you feeling any better than you were yesterday? Are you okay?’

For Chloe, the sense of solidarity that enabled her to talk about her disease and experience was an important and therapeutic aspect of involvement in the APAB:

With these autoimmune diseases sometimes they’re invisible and so you don’t look like you have anything when you’re walking around. So, you’re carrying a lot of that inside of you. And to be able to talk about your experience just decreases a lot of stress, I think, and creates a common bond, and makes you feel like you’re not alone.

For many participants, mentorship from a more senior member of the APAB was a vital part of the nurturing solidarity that helped them adapt to the mindset and orientation of a patient research partner. Irene said that being mentored while she was part of a study meant ‘everything’ to her, helping her to understand that while a particular piece of research might not help her directly it could help others in the future.

The importance of solidarity extended beyond the APAB. Many participants noted that the researchers at Arthritis Research Canada appreciated the contribution made by the patient partners and supported them in other forums. Phoebe recalled being on another panel, which included one of the researchers she had worked with:

I was the only consumer on a panel of doctors, and I raised an issue. And one of the doctors … dismissed it completely and [the researcher] called him on it right at the table and said, ‘Well, I happen to agree with what Phoebe is talking about and let me expand on what I think that she’s saying’.

### Reflection and Reorientation

Many participants explained how putting the negative experience of illness into the service of a positive aspect of their lives had been empowering. As Sarah explained:

It’s a disease that kind of disempowers you and maybe being involved [in the APAB] empowers you or gives you some of that empowerment back … you do see some of the good things that people are doing and you realize … maybe I should be doing a little bit more.

This positive aspect was an outward-looking, civic-minded orientation toward their illness. Irene expressed how joining the APAB was a positive experience during a difficult period:

They’re such a good group and you’re there for a common purpose so [I found myself] binding to the group very easily. It was something else to focus on aside from being sick … I was fairly newly diagnosed and there are so many struggles psychologically that it was just something positive aside from all the negative, the health stuff.

Julie, a long-serving member of the APAB, recognized the transformations that patients undergo during their time on the APAB and the importance of the ongoing recruitment of new members:

On the APAB board you need to nurture both naïve or new people, as well as have the expert patient. You need to work together and be constantly renewing in order to keep the cycle going, because eventually that naïve patient is going to shift and be the expert patient and then they can’t bring that perspective of the regular public to what we’re doing, so it’s a constant renewal.

Lori spoke of the need for a diverse membership on the APAB:

We need a balance, absolutely, because if everybody’s an ‘expert’ then we’re not really digging as deep as we should. Currently, APAB doesn’t represent all socio-economic groups or education levels. That can be a problem especially since we see ourselves as the voice of the patient. Which patient?

Participants did feel a good effort was being made toward representing the diversity of different kinds of arthritis, including various ‘orphan diseases’ on the advisory board.

But other aspects of diversity – such as gender imbalance, ethnic diversity, and diversity of socioeconomic status – were cited by participants as continuing problems which the advisory board should work to address. David, the only man on the advisory board at the time of this study, spoke about the ‘elephant in the room’ of gender imbalance:

I need a little more of the male perspective to be present at the committee table … You know, I can talk about the male perspective but I’m only one guy. There are other men with viewpoints that could add a valuable voice to the conversation … It’s not necessarily tremendously different, it’s just an additional way of looking at the issues and the concerns and the experiences.

In Lori’s and David’s quote alike, we can see the journey of being a patient, or ‘patienthood’ as we have called it, transcending what is usually understood as the responsibilities of being a patient.

## Discussion

In this paper, we sought to describe a connection between the ontologies of patienthood and its practical goals. We examined the process where the *patient perspective* becomes articulated and mobilized as a *patient voice*. It is through this process that the phenomenon of civic patienthood emerges – that is, a patienthood that transcends the individual, bodily concerns of patients themselves and manifests an organized collective will.

Civic patienthood is a social infrastructure for collective action, built upon shared purpose, driven by hope, and grounded in patient experience. It is the name we give to a phenomenon that broader intellectual efforts – such as patient-oriented research, integrated knowledge translation, patient and public involvement in research – all aim to cultivate. It is not a stated framework of values, but an organic approach to solving the problems of illness that is emergent from patient experience.

We see civic patienthood motivated largely by a civic-minded concern for the collective betterment of the experience of illness that research and patient and public engagement within research could achieve. In our interviews, patient partners spoke in altruistic terms about what they aimed to achieve in their involvement as partners in research. This mode of patienthood was characterized by an interest in institutional change and improvement along the lines of inclusion, equity, equality, and better health outcomes. The ‘ethical horizon’ of civic patienthood is one that transcends individual interests to address collective problems in need of collective solutions.

Patient advocacy organizations engage in forward-looking acts of collective responsibility.^27^ The narrow construct of the patient built around the purposes of clinical medicine goes through a two-fold transformation: (1) patient experience is reintegrated as a kind of reflective or skeptical discourse to register discontent; and (2) those individual desires to be understood as a whole person rather than just a patient coalesce into a collective, civic mode of patienthood. In gathering together a collective of patients, the APAB provided an avenue through which a broadened horizon of patienthood could articulate collective concerns. Involvement in the APAB helped generate and give voice to a patienthood that transcends the patient.

Our analysis points to how actions of group solidarity, civic discourse, and the work of collective organization generate new, broader horizons among those involved. The experiences of learning, socializing, and overcoming were transformative, and facilitated by thoughtful mentorship from others in the APAB. Our participants’ interest in helping others was a practical one. Reflecting on their involvement in the APAB led many participants to describe a re-orientation of how they express their patienthood. This re-orientation broadly described an understanding that there are limits to what can be achieved in trying to help oneself in the face of a problem affecting many. From a sociological perspective, the solidarity present on the APAB is reducible neither to altruism nor self-interest. In addressing the structural and systemic problems that may limit the involvement, participants like Julie and Lori demonstrated how becoming a patient partner means participating in an ongoing civic discourse. This is an engagement that turns outward, toward the world and the public as a collective. Though illness, as Heather so memorably put it, ‘stands as the guardian at the gate’ shaping the patient’s experience of the world, a civic orientation towards illness opens new avenues toward addressing the ensuing problems.

These findings resonate with research on the motivations of people to become human research subjects in health research.^22^ That is, a public-oriented sense of responsibility is often intermingled with motivations such as cure-seeking and health consumerism. In this sense, one person can engage in many different modes of patienthood, differing according to how they are relating to their illness.

Not only is the APAB a space for expanding awareness through the process of patient-oriented research, but it also gives patients a channel through which they can articulate their interests *as patients*. In representing a horizon of ethical concern beyond an individual sense of responsibility or interests, APAB was also a living collective project in which our participants invested their hope. We see in the narratives of our participants that their socialization into their role as patient partners involves the incorporation of a collective ethical horizon into their patienthood. This socialization is helped by the APAB, but ultimately has much to do with the patient partner’s journey.

The patient journey includes every aspect of their lives. In the strict medical sense, the patient’s role is to become a subject seen through the filter of medical knowledge. Though often necessary for clinical medicine, this filter labels much of the patient experience irrelevant to the practical concerns of treatment and care. This ‘excess’ of experiences of illness often needs to be expressed elsewhere. An individual patient partner expressing a ‘patient perspective’ in the context of research is one way to bring this ‘excess’ experience created by patienthood back into public discourse. A general kind of skepticism that emerges from patients’ experiences can be organized as feedback into the larger system of official and institutional discourses on health. Patients as a category are a multitude that defies representation through the perspective of a lone individual.^15^

Some scholars^13,16,19,23^ have sought to locate the ontological ground that the concept of ‘the patient experience’ occupies. Williamson^16^ contends that patient engagement has essentially the ontology of an emancipatory political project – as such it requires clarity of purpose, lest it be co-opted and rendered meaningless. Tritter^13^ provides a framework that detailed the differences between patient engagement geared towards collective or individual forms of problem-solving. It is largely Tritter’s framework that we followed into the basic insight that the ontologies of patienthood are multiple, purpose-driven, and can transcend individual patients. Rowland et al.^19^ provide ample evidence that resonates with our own findings, also demonstrating the multiple ontologies (‘modes’, in our words) of patienthood brought to bear in the narratives of patient research partners.

Crucial to achieving clarity around the purposes of patient engagement is understanding which set of ethical concerns are being addressed when we speak about ‘patients’ and ‘patient experience’. Madden and Speed^28^ warn that though the emancipatory project of patient and public involvement in research does hold potential to address many issues in health systems, its ability to do so is circumscribed if the contexts and histories in which it exists are not adequately considered. In putting forth the concept of civic patienthood, we offer a view of these ‘emancipatory projects’ as practical rather than ideological phenomena. We see this as a step in putting a more fully elucidated concept of patient experience at the center of a conversation that ostensibly revolves around it.

Civic patienthood is driven by hope, and that hope is carried forward by people Rose and Novas^29^ refer to as ‘ethical pioneers’ – those who bring issues of patienthood into the civic arena. These pioneers expand the horizons of our thinking about collective ethical responsibilities towards the experience of illness in society. One does not need be a patient to fulfill such a role – our participants gave much credit to the leadership of researchers and clinicians in helping to build the space and capacity for what we call civic patienthood. It is one thing to have skepticism about general and specific approaches to the problems of health and illness – it is entirely another to have an avenue through which one can act on behalf of those concerns. Civic patienthood should be understood primarily as the activity of creating and maintaining those avenues of expression and mobilization; and secondarily as the values, processes, and culture that are generated by this activity. In this formulation civic patienthood is not the end point or ideal, but rather the social infrastructure required to build the capacity to organize and pursue the interests of a collective of patients, whatever those may be.

## Limitations

There are two main limitations to this study. First, the dataset is now around seven years old. Although the fundamental forces our research has revealed are likely to still be in play, it is nevertheless the case that this research does not capture any effects of more recent events. An obvious example is the COVID-19 pandemic, but there may well be other events that have affected the actions and perceptions of our participants.

Second, this research is confined to one patient advocacy board in one country. This potentially limits the generality of our findings. Indeed, the APAB itself is aware that it does not have a diverse membership, lacking representatives from many socioeconomic groups or education levels. Further, the membership is overwhelmingly female. It would be instructive to undertake further research with other patient partner groups, ideally internationally, to see whether their experiences and demographics mirror those of the APAB.

## Conclusions

Our study has put forth a new lens through which we can view the activities of patient engagement in research. But civic patienthood is merely one species of patienthood. Our larger task is to understand how civic patienthood relates to other forms of patienthood that take different approaches to health and illness. For example, there is rich empirical and theoretical ground for elucidating the relationship between the patienthood of clinical medicine and civic patienthood.

Treating these other modes of patienthood as patterns of problem-solving built upon a foundational logic provides a basis to trace how those patterns coalesce into real-world institutions and cultures. Such research could help generate a strategic understanding of how the civic mode of patienthood evident in patient engagement in research can feasibly contribute to system change.

## Supplemental Material

Supplemental Material - A patienthood that transcends the patient: An analysis of patient research partners’ narratives of involvement in a Canadian arthritis patient advisory board Supplemental Material for A patienthood that transcends the patient: An analysis of patient research partners’ narratives of involvement in a Canadian arthritis patient advisory board by Graham G Macdonald, Jenny Leese, Alison M Hoens, Sheila Kerr, Wendy Lum, Lianne Gulka, Laura Nimmon and Linda C Li in Journal of Health Services Research & Policy
