# Supplementary material for: A patienthood that transcends the patient: An analysis of patient research partners’ narratives of involvement in a Canadian arthritis patient advisory board
Source: J Health Serv Res Policy. 2023 Aug 26;29(1):22–30. doi: 10.1177/13558196231197288 (PMC10729530; doi:10.1177/13558196231197288)

Online Supplement

S1: About Us Study interview guide

Introduction: The purpose of today's interview is to understand your perspectives of engaging in research. Before we begin, do you have a pseudonym that you would like to use for this interview? Do you have any questions or concerns? Do I have your permission to turn on the voice recorder?

1. **To begin, please tell me about yourself.**

Probes:

- Tell me a bit about your health.
- Please walk me through how you first became involved with ARC's Arthritis Patient Advisory Board.
- What role(s) have you played in research?

2. **How easy or difficult have you found being involved in research? Please elaborate.**

Probes:

- What do you like or dislike about being involved in research?
- Thinking about your experiences, could you tell me what helps you take part in research?
- Have you found there are any downsides to being involved in research? Could you give an example?
- Can you describe what leads you to continue to be involved in research?
- Can you talk me through what led you to leave APAB? [Emeritus APAB members only]

3. **Could you describe your interactions with researchers at ARC?**

Probes:

- To what extent do researchers partner with you in research?

- To what extent do you feel that your contributions are valued by researchers?
- Have there been any changes in your interactions with researchers over time? Please elaborate.

4. **Could you describe what you think is/has been important for APAB's development?**

Probes:

- Thinking back, what was important for APAB's development in the early years? Has this changed over the years? [More Experienced Members only]
- What can/does/should APAB do that no other organization does or can do as well?
- Can you describe where APAB has most impact?
- Could you describe what you like or dislike about the culture within APAB?
- Can you describe what you like or dislike about how APAB operates?
- Can you talk me through how APAB has secured funding?
- How do you hope to see APAB develop in the short-term/long-term?
- To what extent does ARC support you to engage in research?

5. **Are there any benefits to being involved in research at ARC? If so, please elaborate.**

Probes:

- To what extent do you feel that your involvement is recognized?
- Has being involved in research affected the way that you manage other priorities/manage your health? If so, to what extent?

Thank you very much for your time. We really appreciate you sharing your experiences.

Journal of Health Services Research & Policy

A patienthood that transcends the patient: An analysis of patient research partners' narratives of involvement in a Canadian arthritis patient advisory board

Macdonald GG, Leese J, Hoens AM, et al.

S2: Timeline of iterations of this research

This diagram is intended to provide a bird's eye view of five iterations of this project, showing for each iteration: (Top cell) the general thrust of the work at the time; (Bottom right cell) themes of memos and feedback; and (Bottom left cell) the resolution or product resulting from reflection on the feedback and memos.

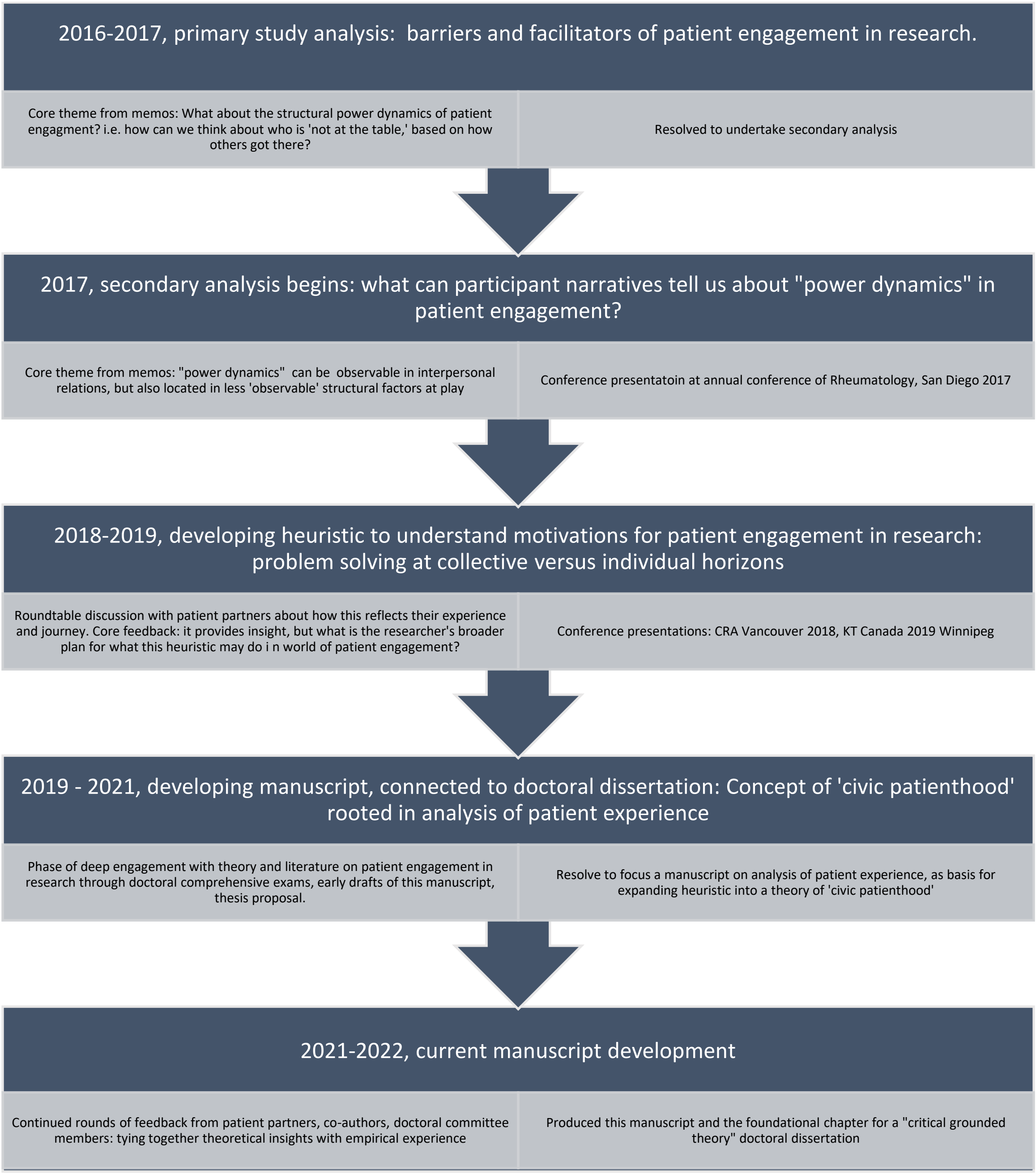

Supplement: Supplemental Material - A patienthood that transcends the patient: An analysis of patient research partners’ narratives of involvement in a Canadian arthritis patient advisory board [file sj-pdf-1-hsr-10.1177_13558196231197288.pdf]
